# Biomarker array for prediction of acute kidney injury after percutaneous coronary intervention for patients who had acute ST segment elevation myocardial infarction

**DOI:** 10.1007/s00380-023-02330-0

**Published:** 2023-11-13

**Authors:** Amr Alkassas, Yasser Elbarbary, Mohammed H. Sherif, Shaimaa B. El-Saied, Rasha Y. Hagag, Mohammed Elbarbary

**Affiliations:** 1 Department of Cardiology, Faculty of Medicine, Tanta University, Tanta, 13111 Gharbia Egypt; 2 Department of Internal (General) Medicine, Faculty of Medicine, Tanta University, Tanta, Gharbia Egypt

**Keywords:** Percutaneous coronary intervention, Acute myocardial infarction, Acute kidney injury, Fatty acid-binding protein, Neutrophil/lymphocyte ratio

## Abstract

Acute kidney injury (AKI) is a common complication after Percutaneous Coronary Intervention (PCI) for ST segment elevation myocardial infarction (STEMI) and is associated with poor outcomes. AKI is diagnosed by the dynamic change of serum Cr, but it could not predict AKI. This study aimed to evaluate a biomarker array that may fulfill this shortage. Setting: Cardiology Department, Tanta University Hospital. Design: Prospective interventional study included 280 acute STEMI patients who underwent emergency PCI. Serial samples of blood and urine were obtained at the time of admission to the hospital (T0) and PCI unit (T1) and at 12 h and 72 h (T12 and T72) after coronary revascularization to estimate levels of serum Cr, creatine phosphokinase, and heart-type fatty acid-binding protein (H-FABP) and calculation of neutrophil/lymphocyte ratio (NLR) and urinary liver-type FABP (L-FABP). AKI was diagnosed according to the recommendations of the European Renal Best Practice as the times of increased serum Cr concerning baseline level. 85 patients developed AKI. Regression analyses defined a high NLR ratio in the T0 sample as the most significant predictor for early AKI diagnosed at T1 time, while high NLR and serum H-FABP levels in T1 samples as the significant predictors for AKI defined at T12 time. However, high urinary L-FABP levels in T12 samples and high NLR are significant predictors for AKI at T72 time. Combined estimations of serum H-FABP and urinary L-FABP with the calculation of NLR could predict the oncoming AKI and discriminate its pathogenesis. The study protocol was approved by the Local Ethical Committee at Tanta Faculty of Medicine by approval number: 35327/3/22. For blindness purposes, the authors will be blinded about the laboratory results till the end of 72 h after revascularization and the clinical pathologist will be blinded about the indication for the requested investigations.

## Introduction

Acute kidney injury (AKI) is marked by deterioration of renal function within hours or days and is manifested by minor rises in serum creatinine (Cr) up to anuric renal failure needing renal replacement therapy (RRT) [1].

There are multiple risk factors for AKI development in patients who had acute coronary syndrome (ACS) especially the presence of chronic kidney disease, diabetes mellitus, old age, anemia, and dehydration [2]. Moreover, systemic hypoperfusion in patients with acute myocardial infarction (AMI) causes renal ischemia secondary to the acute left ventricular systolic failure leading to the development of AKI [3].

Contrast-induced AKI (CI-AKI) was defined as an increased serum Cr level by ≥ 0.5 mg/dl, or ≥ 25%, over the baseline level within 72 h after a contrast material (CM) administration [4]. CI-AKI is mostly the third most common cause of hospital-acquired AKI [5] and ACS patients have a threefold higher risk of developing CI-AKI [6]. Unfortunately, CI-AKI is associated with poor outcomes with prolonged hospitalization and increased morbidity and mortality rates [7].

Heart-type fatty acid-binding protein (H-FABP) is a low molecular weight cytosolic protein, which is primarily expressed in myocardial tissue and functions as the principal transporter of long-chain fatty acids in the cardiomyocyte [8]. Under normal conditions, H-FABP is not present in plasma or interstitial fluid, but is released into the blood upon cardiac cellular injury within 2 h, peaks at about 4–6 h, and returns to its baseline level in 20 h [9], so could be used as an early marker of AMI due to its high sensitivity, specificity and prognostic value [10]. Liver-type fatty acid-binding protein (L-FABP) is a 14kDa protein, which is expressed in the proximal tubular epithelial cells as a carrier of short-chain fatty acid to be transported to and β-oxidized at the mitochondria or peroxisomes [11]. L-FABP is more closely associated with tubular than glomerular dysfunction and is detected in urine only during tubular injury [12].

## Objective

The ability of estimated biomarkers' levels during 72 h after admission of AMI patients to the Emergency Department to predict AKI development and to discriminate its underlying etiology was investigated.

### Setting

Cardiology Department, Faculty of Medicine, Tanta University.

### Design

Prospective comparative interventional study.

### Patients

All patients who arrived at the emergency department from March 2021 till January 2022 with manifestations suggestive of AMI were clinically evaluated for demographic data, smoking, history of previous attacks, maintenance on fibrinolytic drugs, and associated medical conditions, or cardiovascular surgeries. Then, patients underwent complete cardiac examination including ECG to evaluate presence or absence of ST segment elevation and echocardiography to evaluate the ventricular functions.

### Diagnostic definitions

1. STEMI was diagnosed according to the 2017 ESC Guidelines for the management of AMI in patients presenting with typical angina pectoris-like manifestation with elevated serum creatine kinase and cardiac Troponin I. ST segment elevation was defined as at least 0.2 mV elevations in ≥ 2 contiguous precordial leads or at least 0.1 mV elevations in the limbs lead [13].
2. CKD was defined according to the Chronic Kidney Disease Epidemiology Collaboration (CKD-EPI) serum Cr-based equation for estimating glomerular filtration rate (GFR) as at-admission eGFR of ≤ 60 ml/min/1.73 m^2^ [14].
3. AKI was diagnosed according to the recommendations of the European Renal Best Practice [15] for diagnosis and severity staging of AKI as Stage I if there was an increased serum Cr level by 1.5–1.9 times the baseline level or if it was increased by > 0.3 mg/dl or if urine output (UOP) was < 0.5 ml/kg/h for a 6-h block; Stage II if serum Cr level was increased by 2–2.9 times the baseline level or if UOP was < 0.5 ml/kg/h for two successive 6-h blocks; and Stage III if there was increased serum Cr level by > 3 times the baseline level or if UOP was < 0.3 ml/kg/h during > 24 h, anuria for > 12 h or if RRT was initiated.
4. The probable patients' prognosis was predicted according to Killip's classification with no clinical signs of heart failure indicating class I, presence of rales or cracks in lung and S3 gallop, and elevated jugular venous pressure defining class II patients, presence of manifestation of acute pulmonary edema indicates class III, while manifestations of cardiogenic shock with blood pressure < 90mmHg and evidence of low cardiac output indicates class IV [16].

### Exclusion criteria

The exclusion criteria included medical reperfusion using fibrinolytic drugs, provisional diagnosis of non-ST segment elevation myocardial infarction (NSTEMI), diabetic nephropathy, or maintenance on RRT. Patients who also had history of receiving nephrotoxic, immunosuppressing, and chemotherapeutic drugs were also excluded. Patients of Killip's classes II–IV who had circulatory impairment were considered as high-risk patients, and patients who refused to undergo percutaneous coronary intervention (PCI) were excluded and transferred to department for receiving the appropriate treatment. During the procedure, patients who developed cardiogenic shock or required mechanical ventilation were also excluded from statistical analyses.

### Inclusion criteria

Patients with a provisional diagnosis of ST segment elevation myocardial infarction (STEMI) who did not receive medical fibrinolysis, accepted to undergo emergency PCI, and free from exclusion criteria were enrolled in the study.

### Laboratory workup

#### Sample collection and processing

At the time of admission to ED, urine and blood samples were obtained and prepared to undertake the assigned investigations:

1. Urine samples: Random midstream urine sample was collected in a sterile tube and immediately centrifuged at 3000 rpm for 20 min. The supernatants were carefully separated into two parts:
  - The first part: 1 ml of supernatant was collected in 1.5 ml sterile dry tubes and preserved at − 20 °C till be ELISA assayed for urinary L-FABP levels.
  - The second part: 3 ml of supernatant was collected in a clean dry tube for measurement of urinary Cr.
2. Blood samples (5 ml) were obtained under complete aseptic conditions from the antecubital vein; two blood samples were obtained:
  The 1st blood sample was divided into two parts, one was collected in a sodium fluoride (2 mg sodium fluoride/ml blood) containing tube to prevent glycolysis for estimation of blood glucose levels and the other part was collected in tri-potassium ethylenediaminetetraacetate (K3-EDTA) containing tube for complete blood count including a differential leukocytic count for calculation of neutrophil/lymphocyte ratio (NLR).
  The 2nd blood sample was collected in a plain tube and blood was allowed to clot in a warm water bath at a temp of 37 °C for 5 min and then centrifuged at 5000 rpm for 2 min to separate serum. The resultant serum sample was divided into two parts: one was used for estimation of serum Cr and creatine phosphokinase (CPK) and the 2nd part was collected in a clean Eppendorf tube and stored at − 20 °C for ELISA estimation of human H-FABP

### Investigation

1. Blood glucose level using glucose oxidase method [17].
2. Serum creatine phosphokinase uses a method, which is based on the reaction of creatine, formed enzymatically from creatine phosphate and ADP, with *p*-nitrophenylglyoxal under alkaline conditions to produce a colored product that absorbs maximally at 480 nm [18].
3. Serum creatinine using the Jaffe-based creatinine–picrate formation in an alkaline medium. [19, 20]
4. Serum human H-FABP was measured with the enzyme-linked immunoassay (ELISA) kit (catalog no. ab243682 Abcam Inc., San Francisco, USA) by quantitative sandwich enzyme immunoassay technique [21].
5. Urinary creatinine (uCr) was measured with a creatinine (urinary) colorimetric assay kit (catalog no. 500701, Cayman Chemical, Ann Arbar, MI, USA) by Jaffe’s reaction method [22].
6. Urinary L-FABP was measured with the enzyme-linked immunoassay (ELISA) kit (catalog no. ab2218261 Abcam Inc., San Francisco, USA) by quantitative sandwich enzyme immunoassay technique. The total urinary L-FABP levels were normalized to the estimated Cr concentration to determine urinary L-FABP/Cr (µg/gCr) ratios [11].

### Management protocol

- According to the 2017 ESC Guidelines for the management of AMI in patients presenting with ST segment elevation, the time of diagnosis of STEMI during ECG examination was considered as the zero time if the patient had arrived directly to the PCI unit within < 10 min after pain sensation and PCI was undertaken after diagnosis. The maximum allowable time for primary PCI intervention is ≤ 120 min.
- Serial estimation of serum Cr levels for diagnosis of AKI according to the ERBP recommendations [15], with an estimation of serum CPK and calculation of N/L ratio. Multiple blood samples were obtained at the time of admission to the emergency department to obtain baseline data (T0 sample), at the time of admission to the PCI unit, and before CM injection for evaluation of the effect of ischemia secondary to AMI on kidney functions (T1 sample), at 12 h after revascularization (T12 sample) for evaluation of the effects of revascularization and immediate effects of CM on kidney functions and 72 h (T72 sample) after CM injection for diagnosis of CI-AKI.

### Study outcomes

1. **The primary outcome** is the ability of estimated serum and urinary biomarkers levels and NLR to discriminate STEMI patients liable to develop AKI.
2. **The secondary outcomes** include:
3. The ability of the estimated biomarkers' levels to differentiate between patients who developed STEMI-induced AKI and those who developed CI-AKI.
4. The determination of the predictability of using more than one marker to achieve outcomes.

### Sample size

The sample size was calculated by the G*Power (Version 3.1.9.2) [23]. A previous study of the relation between the L-FABP level and the development of AKI during AMI attack reported that the median level of L-FABP in No-AKI patients was 1 (range 0.5–2.8 ng/ml), while in AKI patients, the median value was 2.1; range 0.67–6.3 ng/mL [24]. Considering the null hypothesis was the absence of difference in the L-FABP level between AKI and No-AKI groups and considering the previously documented AKI incidence rate during interventional cardiology which was 29.5% [24] and 36% [25], so to achieve a similar incidence of AKI, using α error 5% and a power of 80%, and considering the required sample size for AKI group must be 85 patients, the total patients' number must be 250 to get an AKI incidence of 34%.

## Results

**Fig. 1:**
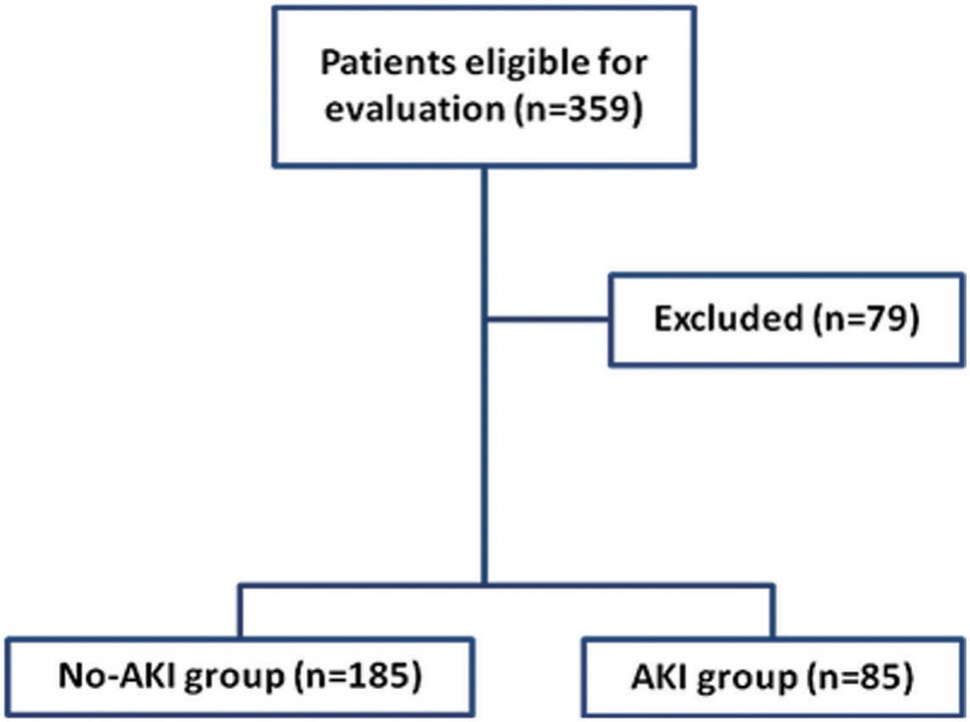
Patients’ flowchart

During the study duration, 359 patients were eligible for evaluation, 79 patients were excluded for not fulfilling the inclusion criteria, and 280 patients were enrolled in the study. Serum Cr levels estimated at the time of hospital admission (T0 Sample) showed non-significant differences between patients. However, serum Cr levels were significantly higher in T1, T12, and T72 samples compared to levels estimated in T0 samples. Estimated serum Cr levels in samples obtained at T1, T12, and T72 times were increased in samples of 13, 35, and 37 patients, respectively, up to the AKI diagnostic levels, and these 85 patients were grouped as AKI group. In the same samples of the remaining 195 patients, the times of increase in serum Cr were not diagnostic for AKI (No-AKI group) and significantly lower in comparison to that of AKI patients (Table 1, Fig. 1). According to times of increase in serum Cr levels concerning T0 levels, there were 40 patients of AKI grade I, 41 patients of AKI grade II, and 4 patients of AKI grade III (Table 1). Revision of enrollment data of studied patients after grouping showed non-significant differences between T0 data of patients of both groups as shown in Table 2.

**Table 1:** Results of the serial estimations of serum Cr in studied patients

Sampling time	Total patients	No-AKI	AKI	*P*-value
T0				
Number (%)	280 (100%)	195 (69.64%)	85 (30.36%)	
Level (mg/dl)	0.77 ± 0.24	0.75 ± 0.25	0.81 ± 0.23	**0.064**
T1				
Number (%)	280 (100%)	267 (95.36%)	13 (4.64%)	
Level (mg/dl)	0.87 ± 0.32	0.84 ± 0.26	1.53 ± 0.62	** < 0.0001**
P1	**0.0002**			
Times of increase*	1.07 (1.05–1.13)	1.07 (1.05–1.12)	1.7 (1.54–1.98)	** < 0.0001**
T12				
Number (%)	280 (100%)	232 (82.86%)	48 (17.14%)	
Level	1 ± 0.48	0.85 ± 0.25	1.77 ± 0.59	** < 0.0001**
P1	** < 0.0001**	**0.54**	**0.195**	
Times of increase*	1.16 (1.11–1.28)	1.15 (1.1–1.21)	1.86 (1.75–1.98)	** < 0.0001**
T72				
Number (%)	280 (100%)	195 (69.64%)	85 (30.36%)	
Level	1.13 ± 0.54	0.89 ± 0.24	1.68 ± 0.62	** < 0.0001**
P1	** < 0.0001**	**0.154**	**0.543**	
Times of increase*	1.24 (1.16–1.73)	1.18 (1.14–1.25)	2.01 (1.78–2.21)	** < 0.0001**
Time	T1	T12	T72	Total
AKI grade				
No-AKI	267 (95.4%)	245 (87.5%)	243 (86.8%)	**195 (69.7%)**
AKI				
Stage I	10 (3.6%)	27 (9.6%)	3 (1%)	**40 (14.3%)**
Stage II	3 (1%)	5 (1.8%)	33 (11.8%)	**41 (14.6%)**
Stage III	0	3 (1%)	1 (0.4%)	**4 (1.4%)**
Total	**13 (4.6%)**	**35 (12.4%)**	**37 (13.2%)**	**85 (30.3%)**

*P*-value indicates the significance of difference between both groups at cutoff point of *P*< 0.05

Data are shown as mean, standard deviation, numbers, percentages, median, and interquartile range*; AKI: Acute kidney injury; T0: time of hospital admission; T1: Time of PCI admission; T12: 12 h after revascularization; T72: 72 h after revascularization; Times of increase = times of increased level of the current estimation concerning level estimated in T0 sample; *P*-value indicates the significance of the difference between AKI and No-AKI patients; P1 value indicates the significance of difference versus the preceding estimation; *P* < 0.05 indicates the significant difference; *P* > 0.05 indicates the non-significant difference

**Table 2:** Enrollment data of studied patients categorized according to development of AKI at 72 h after revascularization

Variable	Group	No-AKI (*n* = 195)	AKI (*n* = 85)	*P*-value
Age (years)		61.8 ± 6.8	63.6 ± 8.7	**0.063**
Gender	Males	123 (63.1%)	61 (71.7%)	
	Females	72 (36.9%)	24 (28.3%)	**0.159**
Smoking	Ex-smokers	60 (30.8%)	23 (27.1%)	
	Current smokers	51 (26.2%)	21 (24.7%)	**0.713**
	Non-smokers	84 (43%)	41 (48.2%)	
Family history of CAD		26 (13.3%)	16 (18.8%)	**0.237**
Associated risk medical conditions	Diabetes mellitus	51 (26.2%)	28 (32.9%)	**0.071**
	Hypertension	109 (55.9%)	53 (62.4%)	**0.315**
	Hyperlipidemia	84 (43.1%)	44 (51.7%)	**0.179**
Current medications	Statins	41 (21%)	27 (31.7%)	**0.054**
	Aspirin	165 (84.6%)	67 (78.8%)	**0.237**
	B-blockers	147 (75.4%)	71 (83.5%)	**0.131**
	ACE inhibitors	153 (78.5%)	64 (75.3%)	**0.559**
	ARB	35 (17.9%)	18 (21.2%)	**0.526**
Time-lag till hospital arrival (h)		4.7 ± 4.4	5.83 ± 4.2	**0.053**
Ejection fraction (%)		57 ± 8.3	56 ± 8	**0.373**
Baseline cardiac markers' levels	Troponin I (ng/ml)	6.3 ± 1.76	5.85 ± 2.3	**0.073**
	CPK (U/L)	1349.5 ± 543.5	1472.7 ± 430.3	**0.065**

*P*-value indicates the significance of difference between both groups at cutoff point of *P*< 0.05

Data are shown as mean, standard deviation, numbers, percentages; AKI: Acute kidney injury; CAD: Coronary artery disease; ACE: Angiotensin-converting enzyme; ARB: Angiotensin II receptor blockers; CPK: Creatine phosphokinase; *P*-value indicates the significance of the difference between both groups; *P* < 0.05 indicates the significant difference; *P* > 0.05 indicates the non-significant difference

Serum CPK levels were increased in T1 samples significantly more than in T0 samples but decreased in T12 samples significantly compared to levels estimated in T0 and T1 samples. Serum levels of CPK were non-significantly higher in T0 and T12 samples but significantly higher in T1 samples of AKI than in No-AKI patients.

Serum levels of H-FABP showed a progressive increase in T1 and T12 samples with a significant difference between levels estimated in T0 samples and between levels estimated in T1 and T12 samples. However, the difference between AKI and No-AKI patients was non-significant in T0 samples but was significant in T1 and T12 samples.

The calculated NLR was significantly higher in patients of the AKI group compared to those of the No-AKI group in all samples and significantly higher in each sample in comparison to the preceding samples of AKI patients. On contrary, in samples of No-AKI patients, the ratio was significantly higher in T1 and T12 samples than in T0 and T72 samples with a non-significantly higher ratio in T1 than in T12 samples.

Estimated urinary levels of L-FABP in T1 samples showed a non-significant difference in comparison to levels estimated in T0 samples of patients in both groups. In T1 samples, urinary L-FABP levels were significantly higher in samples of AKI than in No-AKI patients. Urinary L-FABP levels estimated in T12 samples were significantly higher compared to levels estimated in the preceding samples with significantly higher levels in samples of AKI than in No-AKI patients. At 72 h after revascularization, urinary L-FABP levels were higher in samples of AKI patients but decreased in samples of No-AKI patients in comparison to levels estimated at T1 and T12 and with significantly higher levels in AKI than No-AKI samples (Table 3).

**Table 3:** Results of the serial estimations of levels of laboratory parameters

Parameters	Total patients	No-AKI	AKI	*P*-value
Serum CPK (U/L)				
T0	1411.9 ± 575.5	1385.4 ± 627.5	1472.7 ± 430.3	0.244
T1	1842.4 ± 789.2	1771.3 ± 782.5	2005.4 ± 784.9	0.022
P1 value	** < 0.0001**	** < 0.0001**	** < 0.0001**	
T12	816.4 ± 271	810 ± 266.4	830.9 ± 282.4	0.553
P1 value	** < 0.0001**	** < 0.0001**	** < 0.0001**	
P2 value	** < 0.0001**	** < 0.0001**	** < 0.0001**	
Serum H-FABP (ng/ml))				
T0	10.52 ± 3.56	10.33 ± 3.48	10.95 ± 3.7	0.179
T1	13.2 ± 4.64	12.4 ± 4.18	15.06 ± 5.1	< 0.0001
P1 value	** < 0.0001**	** < 0.0001**	** < 0.0001**	
T12	14.87 ± 5.7	14 ± 5.28	16.85 ± 6.16	0.0001
P1 value	** < 0.0001**	** < 0.0001**	** < 0.0001**	
P2 value	** < 0.0001**	** < 0.0001**	** < 0.0001**	
Neutrophil/lymphocyte ratio				
T0	1.46 ± 0.55	1.39 ± 0.47	1.6 ± 0.68	0.0029
T1	1.93 ± 0.59	1.85 ± 0.49	2.13 ± 0.73	0.0002
P1 value	** < 0.0001**	** < 0.0001**	** < 0.0001**	
T12	2 ± 0.49	1.8 ± 0.39	2.47 ± 0.35	< 0.0001
P1 value	** < 0.0001**	** < 0.0001**	** < 0.0001**	
P2 value	**0.502**	**0.759**	**0.0005**	
T72	1.95 ± 0.81	1.45 ± 0.32	3.07 ± 0.33	< 0.0001
P1 value	** < 0.0001**	**0.418**	** < 0.0001**	
P2 value	**0.993**	** < 0.0001**	** < 0.0001**	
P3 value	**0.675**	** < 0.0001**	** < 0.0001**	
Urinary L/FABP (µg/gCr)				
T0	4.1 ± 2.9	4.07 ± 2.85	4.17 ± 3.03	0.801
T1	5.21 ± 3.66	4.89 ± 3.39	5.93 ± 4.14	0.0285
P1 value	0.036	0.072	0.170	
T12	6.2 ± 4.57	5.37 ± 3.65	8.11 ± 5.76	< 0.0001
P1 value	** < 0.0001**	0.0007	0.0004	
P2 value	** < 0.0001**	**0.020**	** < 0.0001**	
T72	8.31 ± 7.22	5.05 ± 3.37	15.79 ± 8.11	< 0.0001
P1 value	**0.074**	**0.486**	**0.055**	
P2 value	** < 0.0001**	**0.965**	** < 0.0001**	
P3 value	< 0.0001	0.778	< 0.0001	

*P*-value indicates the significance of difference between both groups at cutoff point of *P*< 0.05

Data are shown as mean, standard deviation; AKI: Acute kidney injury; CPK: Creatine phosphokinase; H-FABP: Heart-type fatty acid-binding protein; L-FABP: Liver-type FABP; T0: time of hospital admission; T1: Time of PCI admission; T12: 12 h after revascularization; T72: 72 h after revascularization; *P*-value indicates the significance of the difference between AKI and No-AKI groups; P1 value indicates the significance of different levels estimated at T1, T12, and T72 versus T0; P2 value indicates the significance of different levels estimated at T12 and T72 versus T1; P3 value indicates the significance of different levels estimated at T72 versus T12; *P* < 0.05 indicates the significant difference; *P* > 0.05 indicates the non-significant difference

According to the results of linear univariate and multivariate regression analyses, a high NLR ratio in the T0 sample was defined as the most significant predictor for the times of increase in serum Cr in T1 samples and high NLR and serum H-FABP levels in T1 samples as the significant predictors for the times of increase in serum Cr levels in T12 samples. On the other hand, high L-FABP levels in T12 urinary samples and high NLR in blood samples are significant predictors for the times of increase in serum Cr in T72 samples (Table 4).

**Table 4:** Regression analysis of biomarkers' levels estimated as a predictor for the times of increase in serum Cr levels concerning levels estimated in the T0 sample

Item	Statistical analyses	Univariate		Multivariate	
	Variable	β	*P*	β	*P*
T0 levels as predictors for times of increased serum Cr in T1 samples	NLR	0.095	** < 0.001**	0.076	** < 0.001**
	Serum CPK level	0.003	**0.027**	0.045	**0.047**
	Serum H-FABP	0.010	**0.036**	0.012	**0.035**
T1 levels as predictors for times of increased serum Cr in T12 samples	NLR	0.249	** < 0.001**	0.326	** < 0.001**
	Serum CPK level	0.013	**0.013**	0.010	**0.011**
	Serum H-FABP	0.024	**0.002**	0.019	**0.009**
	Urinary L-FABP	0.008	**0.036**	0.007	**0.026**
T12 levels as predictors for times of increased serum Cr in T72 samples	NLR	0.423	**0.035**	0.013	**0.007**
	Serum CPK level	0.027	**0.132**	0.047	**0.376**
	Serum H-FABP	0.045	**0.230**	0.296	**0.182**
	Urinary L-FABP	0.026	** < 0.001**	0.011	** < 0.001**

*P*-value indicates the significance of difference between both groups at cutoff point of *P*< 0.05

*β*: Regression coefficient; NLR: Neutrophil/lymphocyte ratio; AKI: Acute kidney injury; CPK: Creatine phosphokinase; H-FABP: Heart-type fatty acid-binding protein; L-FABP: Liver-type FABP; T0: time of hospital admission; T1: Time of PCI admission; T12: 12 h after revascularization; T72: 72 h after revascularization; *P*-value indicates the significance of β; *P* < 0.05 indicates the significant difference; *P* > 0.05 indicates the non-significant difference

## Discussion

Estimated serum Cr levels in T0 samples were within the normal range but were increased in all patients up to the diagnostic level for AKI in 13, 35, and 37 patients in T1, T12, and T72 samples. AKI cases that were detected at the time of admission to PCI (T1 time) could be attributed to the effect of ischemia secondary to the coronary obstruction that resulted in AMI. In line with this attribution, recent experimental studies detected reduced renal blood flow by about 40% with cardiac ischemia [27].

The development of AKI at 12 h after revascularization (T12 time) may be secondary to the effect of the washed inflammatory cytokines and free radicals in association with cardiac and renal ischemia–reperfusion injury as previously detected in animal models of ischemia–reperfusion AKI [28, 29] or may be due to early-onset contrast-induced AKI (CI-AKI) that was previously detected using an animal model of CI-AKI where microscopic examination assured that the strongest renal damage in CI-AKI existed between 12 and 24 h after CM injection [28]. On the contrary, AKI cases detected at 72 h after revascularization (T72 time) are considered CI-AKI as previously documented [4]. The reported AKI rate was in line with or lower than that reported by previous studies [30–32] evaluated similar condition and detected AKI incidence ranging between 29.6 and 46%.

Statistical analyses defined serum levels of H-FABP in T0 and T1 samples as a significant predictor, while serum levels of CPK despite being elevated in T0 and T1 samples were less significant predictors for increases in serum Cr at T1 and T12 respectively, and could predict increased serum Cr levels at T1 only. These data assured the relation between cardiac ischemia and development of AKI and supported the previous works that reported high sensitivity and specificity of high serum H-FABP to predict AKI [33] and worsening of renal function [34] in the setting of acute heart failure, especially in non-chronic kidney disease patients. A recent study documented that serum level of H-FABP was found to be changed significantly after PCI and can predict AKI with high sensitivity and negative predictive value [35].

The low levels of L-FABP in T0 and T1 urine samples manifest the low-grade early kidney affection as previously documented that kidneys try to preserve L-FABP, which is fatty acid transporter for renal mitochondria [11], but its progressive increase in the subsequent samples indicated renal tubular injury. The detected increase in urinary L-FABP in T1 than in T0 samples indicated the effect of prerenal ischemia secondary to AMI, which induces up-regulation of the expression levels of L-FABP to bind the ischemia-induced lipid peroxidation products and transferring them to urinary spaces [36] and this might explain the reported significant difference in urinary L-FABP levels in T1 samples of AKI and No-AKI patients.

Statistical analyses defined high urinary L-FABP in the T12 sample as a predictor and the T72 sample as a significant discriminator for AKI cases. The obtained data are in hand with the previously reported significant differences between levels of urinary L-FABP estimated at 24 and 48-h after dye injection before cardiac surgery [37] and with the conclusion that estimation of urinary L-FABP levels improves early prediction of AKI in patients hospitalized at medical cardiac intensive care units [38] and after coronary angiography/PCI [39]. Also, these results are in line with the survey study that found cardiologists who introduced pre- and peri-cardiac interventions estimation of L-FABP into patient care were 2.9 times more likely to correctly identify their patients' risk for AKI and were more than twice as likely to treat AKI [40].

The estimated high NLR in T0 and T1 samples indicated its relation with cardiac ischemia and supported the previous data that the NLR is an independent risk factor and a predictor of coronary artery disease (CAD) [41, 42]. Moreover, statistical analyses of NLR in T0 and T1 samples showed the significant predictability of high NLR in early samples for oncoming AKI. These findings assured the reported high predictability of a high systemic immune inflammation index, which equals the NLR multiplied by platelet count, to define patients liable to develop AKI after PCI [43, 44].

Further, high NLR in the T12 sample was defined as a significant predictor for the development of CI-AKI at 72 h after injection. These findings indicated two important points: first, early estimation of NLR could predict early AKI after dye injection and this suggestion went in hand with a previous study, which found high NLR was an independent predictor of CI-AKI in AMI patients and recommended its use to improve on current risk prediction models [4]. Second, the reported progressively increasing NLR up to 72 h after intervention pointed to the inflammatory origin of CI-AKI and assured the results obtained using AKI animal models that inhibition of NLRP3 inflammasome attenuated renal apoptosis and upregulated HIF1A and BNIP3-mediated mitophagy in CI-AKI [45] and the study which detected renal neutrophil and macrophage chemotaxis, accumulation of the lymphocyte antigen 6 complex with pyroptosis, mitophagy, and apoptosis in renal tissues of CI-AKI animal model [28].

Thus, elevated serum levels of H-FABP and high NLR before PCI could predict the development of AKI secondary to myocardial ischemia, while high NLR and urinary L-FABP 12 h after PCI could predict CI-AKI, and high urinary L-FABP levels and NLR 72 h after PCI could define AKI patients.

## Conclusion

The obtained results spotlight the ability of the combined estimations of serum heart-type and urinary liver-type FABP with the calculation of NLR to predict the oncoming AKI and discriminate its underlying pathogenesis.

### Recommendations

The same combination of the studied laboratory variables could be used to evaluate maneuvers to be used to minimize the deleterious effect of CM as pre-procedural hydration and metformin and for evaluating the effect of varied types of dyes.

## Data Availability

Available on request.
